# KMT2D maintains neoplastic cell proliferation and global histone H3 lysine 4 monomethylation

**DOI:** 10.18632/oncotarget.1555

**Published:** 2013-11-03

**Authors:** Changcun Guo, Lee H. Chen, Yafen Huang, Chun-Chi Chang, Ping Wang, Christopher J. Pirozzi, Xiaoxia Qin, Xuhui Bao, Paula K. Greer, Roger E. McLendon, Hai Yan, Stephen T. Keir, Darell D. Bigner, Yiping He

**Affiliations:** ^1^ The Preston Robert Tisch Brain Tumor Center at Duke and Pediatric Brain Tumor Foundation Institute, Duke University, Durham, NC; ^2^ Department of Pathology, Duke University, Durham, NC; ^3^ Department of Surgery, Duke University, Durham, NC; ^4^ Institute for Genome Sciences and Policy, Duke University, Durham, NC

**Keywords:** MLL2, isogenic cell line, gene knockout, enhancer

## Abstract

KMT2D (lysine (K)-specific methyltransferase 2D), formerly named MLL2 (myeloid/lymphoid or mixed-lineage leukemia 2, also known as ALR/MLL4), is a histone methyltransferase that plays an important role in regulating gene transcription. In particular, it targets histone H3 lysine 4 (H3K4), whose methylations serve as a gene activation mark. Recently, KMT2D has emerged as one of the most frequently mutated genes in a variety of cancers and in other human diseases, including lymphoma, medulloblastoma, gastric cancer, and Kabuki syndrome. Mutations in KMT2D identified thus far point to its loss-of-function in pathogenesis and suggest its role as a tumor suppressor in various tissues. To determine the effect of a KMT2D deficiency on neoplastic cells, we used homologous recombination- and nuclease-mediated gene editing approaches to generate a panel of isogenic colorectal and medulloblastoma cancer cell lines that differ with respect to their endogenous KMT2D status. We found that a KMT2D deficiency resulted in attenuated cancer cell proliferation and defective cell migration. Analysis of histone H3 modifications revealed that KMT2D was essential for maintaining the level of global H3K4 monomethylation and that its enzymatic SET domain was directly responsible for this function. Furthermore, we found that a majority of KMT2D binding sites are located in regions of potential enhancer elements. Together, these findings revealed the role of KMT2D in regulating enhancer elements in human cells and shed light on the tumorigenic role of its deficiency. Our study supports that KMT2D has distinct roles in neoplastic cells, as opposed to normal cells, and that inhibiting KMT2D may be a viable strategy for cancer therapeutics.

## INTRODUCTION

One striking theme emerging from recent findings from high throughput sequencing-based cancer genetic studies is that chromatin remodeling and histone modulators are frequently altered and that the resulting aberrations promote cancers. The best examples include inactivating mutations in genes encoding histone lysine methyltransferases KMT2C (also known as MLL3), KMT2D (also known as MLL2/ALR/MLL4), and their associated histone demethylase KDM6A (also known as UTX) [1-17]. The original medulloblastoma exome sequencing led to the discovery of frequent mutations in the histone lysine methyltransferase gene *KMT2D/MLL2* and its homolog *KMT2C/MLL3*, which were not previously linked to cancer [13]. Subsequent comprehensive studies involving larger numbers of well-classified medulloblastoma samples have significantly extended these genetic findings and further confirmed that dysregulation of the KMT2D/KMT2C pathway, including mutations in a KMT2D- and KMT2C-associated demethylase KDM6A, plays an important role in driving various types of human medulloblastomas [14-17]. The identified KMT2D alterations span across the whole gene and were most frequently heterozygous frameshift or nonsense mutations that ablated the carboxyl-terminal methyltransferase enzymatic domain (SET domain). These findings defined this pathway as a bona fide medulloblastoma oncogenic pathway.

In addition to its role in medulloblastoma, subsequent studies have found that *KMT2D* is frequently mutated in other cancers, including 89% of follicular lymphoma and 20%-30% of diffusive large B-cell lymphoma [8, 9]. *KMT2C* is also mutated in colorectal cancer [1, 10]. Additional cancers that have recently been found to be driven by an aberrant KMT2D/KMT2C pathway, with frequencies ranging from 5% to 40%, include renal [2], prostate [18], bladder [5], gastric [11], hepatic [3] and lung cancer [6, 19]. Furthermore, germline *KMT2D* inactivation has recently been found to be the major cause of Kabuki syndrome [20], a rare, congenital pediatric disorder characterized by intellectual disabilities; de novo mutations in *KMT2D* and other chromatin-modifying genes have been associated with congenital heart disease [21]. Collectively, these findings place the KMT2D/KMT2C pathway among the most frequently mutated pathogenic pathways that drive human diseases. The alterations of KMT2D/KMT2C suggest new opportunities for therapeutics and highlight an urgent need to understand their functional mechanism and therapeutic implications.

Modulation of chromatin accessibility through histone methylations is essential in regulating eukaryotic gene transcription. For example, histone H3 lysine 4 (H3K4) methylation by the histone methyltransferase family of genes is associated with active gene transcription and plays an important role in development [22]. Among the four H3K4 methyltransferase genes with similar structural domains, *KMT2A* (formerly named *MLL*) is frequently involved in genetic alterations in acute myeloid and acute lymphoblastic leukemia [23]. Its homologue, *KMT2B* (formerly named *MLL4*), has been found to be overexpressed in breast and colorectal cancer cell lines, although the pathological consequence of its overexpression is unclear [24]. The remaining pair of genes, *KMT2C and KMT2D*, shares a high degree of similarity in their structural domains and biochemical functions. For example, each of them associates with nuclear receptor coactivator 6 (NCOA6, also known as activating signaling cointegrator-2, ASC-2) to form a complex that contains other essential subunits, including a histone demethylase, KDM6A [28-30]. The KMT2C or KMT2D complex has been found to play essential roles as a coactivator for transcriptional activation by nuclear hormone receptors, including retinoic acid receptors, in *Hox* gene transcription, and in regulating adipogenesis [31-35].

Apart from the genetic evidence, the role of KMT2C in tumorigenesis and in cancer progression was first suggested by a *KMT2C* knockout mouse model that developed ureter epithelial tumors [36]. Furthermore, knockdown of *KMT2C* promoted the proliferation of hepatocellular carcinoma cancer cell lines in vitro [3]. In contrast, knockdown of *KMT2D* resulted in reduced cancer cell proliferation and altered adhesion in HeLa cells [29]. This, together with the near absence of homozygous *KMT2D* inactivating mutations in medulloblastoma [16], led us to speculate that the role of the KMT2D deficiency in cancer is more complicated than simply driving tumor cell proliferation. To clarify the role of KMT2D in cancer cells, we employed somatic gene knockout approaches to generate a panel of isogenic KMT2D-deficient human cancer cell lines and determined the impact of a KMT2D deficiency on neoplastic cells. We further characterized KMT2D binding loci to reveal a link between KMT2D, global H3K4 monomethylation, and enhancer elements.

## RESULTS

### KMT2D deficiency affects proliferation of neoplastic cells

Somatic mutations of *KMT2D* were initially identified in medulloblastoma [13], thus we sought to first examine its role in human medulloblastoma cancer cell lines. The gigantic size of the KMT2D polypeptide (5537 amino acids with a molecular weight of ~600 kDa) makes its gain-of-function study by overexpression difficult and inefficient. Since there are no known medulloblastoma cell lines with loss-of-function *KMT2D* mutations (e.g., frameshift mutations), we decided to use a somatic gene knockout strategy. We selected two human medulloblastoma cancer cell lines, D425MED and D283MED, and transiently transfected a pair of *KMT2D*-specific zinc-finger nucleases (ZFNs) to induce double strand break surrounding the codon for amino acid 3685 (Histidine, H3685) of KMT2D. The generation of a double-strand break led to error-prone non-homologous end joining (NHEJ), resulting in frequent mutations of *KMT2D*. Using this pair of ZFNs to mutate *KMT2D* in medulloblastoma cell lines, we introduced nucleotide alterations in one allele of *KMT2D* through a single round of transient nuclease expression. We selected mutants with heterozygous frameshift mutations (e.g., a small deletion of four bases, as shown in Fig. S1A, that made the allele “null”) as they best resembled somatic alterations identified in human cancers. Since the enzymatic SET domain is located at the carboxyl-terminus that was expected to be ablated by the frameshift mutations, it was unlikely the mutant allele would generate truncated polypeptides possessing gain-of-function enzymatic activity. These isogenic heterozygous KMT2D mutant-harboring cell lines were defined as KMT2D-deficient (*KMT2D^+/−^*) derivatives thereafter.

**Figure 1:**
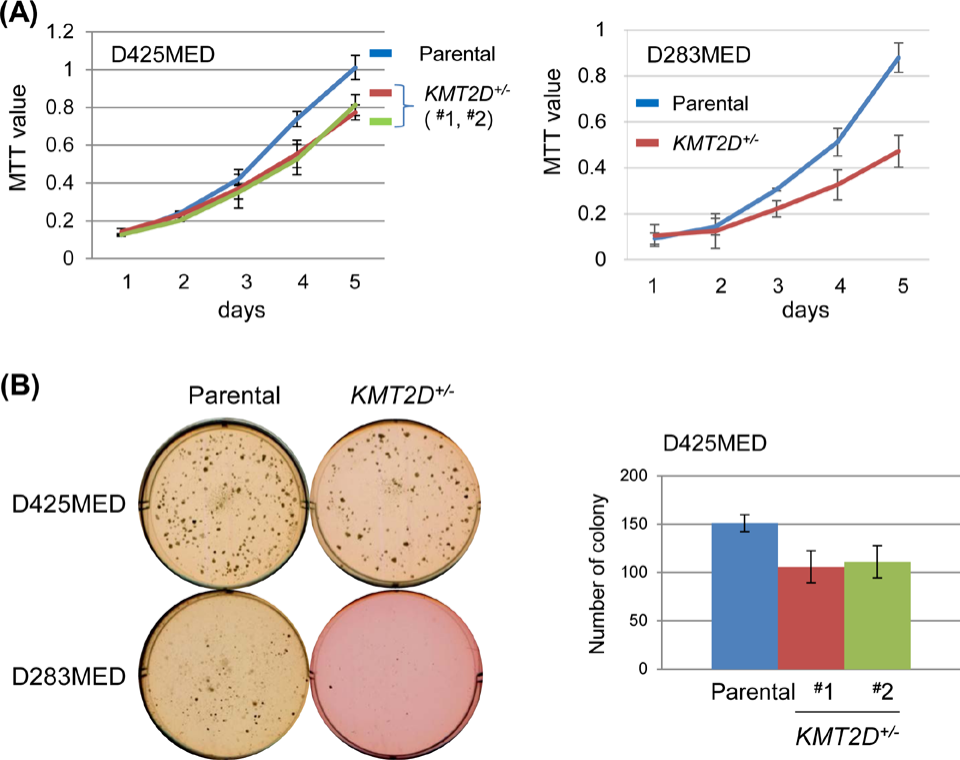
KMT2D deficiency affects proliferation of medulloblastoma cell lines (A) Proliferation of D425MED and D283MED cell lines was measured by MTT assay (for D425MED, each mutant vs. parental: *p*=0.01; for D283MED, *p*<0.01). (B) Cell propagation was measured by colony formation in soft agar. For D425MED cell lines, the number of colonies was counted after a 14-day incubation; representative results (from triplicate wells) from one of three experiments was shown (for each mutant vs. parental: *p*<0.05).

To determine the biological consequence of KMT2D deficiency, we measured the proliferation of KMT2D-deficient cells. As shown in Fig. 1A, in comparison to the parental cells, *KMT2D^+/−^* isogenic cell lines derived from both D425MED and D283MED had slightly, but reproducibly decreased proliferation kinetics under standard culture conditions in vitro. Consistent with a slower proliferation rate, colony formation assays revealed a compromised ability for single *KMT2D^+/−^* cells to form colonies, as reflected by both the numbers and sizes of colonies formed in 6-well plate assays over the course of 12 days (Fig. 1B).

**Figure 2:**
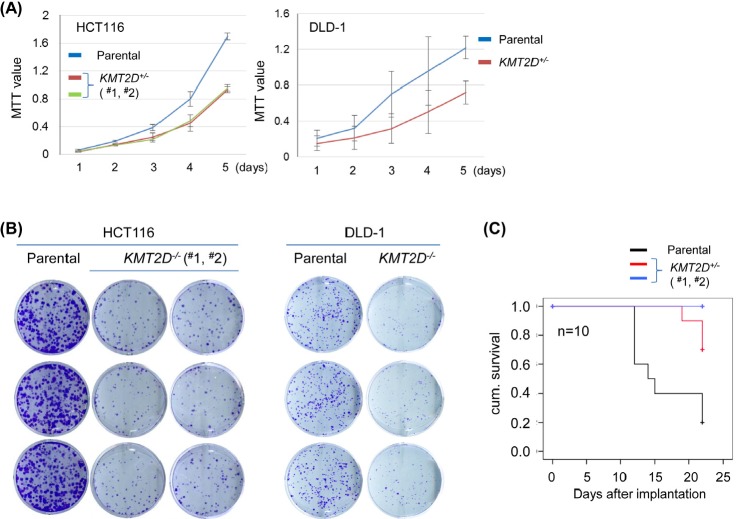
KMT2D deficiency affects proliferation of colorectal cell lines (A) Proliferation of HCT116 and DLD-1 cell lines was measured by MTT assay (for HCT116, each mutant vs. parental: *p*<0.001; for DLD-1, *p*<0.01). (B) Cell propagation was measured by the colony formation assay. (C) Kaplan-Meier survival curve analysis of mice receiving parental or *KMT2D^−/−^* HCT116 cell lines subcutaneously; 10 mice were included for each cell line (mutant #1 vs. parental: *p*=0.01; mutant #2 vs. parental: *p*<0.001).

To decide if the aforementioned effect was specific only to medulloblastoma cell lines, we expanded our experiments to additional cellular contexts. For this purpose, we generated KMT2D-deficient isogenic cell lines derived from colorectal carcinoma lines, including HCT116 and DLD-1. To exclude potential artifacts resulted from particular gene-editing methods, we used different approaches to generate these isogenic cell lines. As we described previously, *KMT2D^−/−^* HCT116 cell lines were obtained by recombinant adeno-associated virus (rAAV)-mediated homologous recombination [35]. The isogenic *KMT2D^−/−^* DLD-1 cell line was obtained by using ZFN- and rAAV-mediated approach sequentially for the first and second allele gene knockout (Fig. S1B). We measured the proliferation of these *KMT2D^−/−^* isogenic cell lines in comparison to their respective parental cell lines. Again, *KMT2D* knockout resulted in attenuated cell proliferation in both HCT116 and in DLD-1 and compromised the ability of the cells to form colonies (Fig. 2A–B). To ascertain that the compromised cell proliferation was not specific only for the in vitro culture condition, we performed in vivo tumorigenicity assays of paired parental versus *KMT2D^−/−^* isogenic HCT116 cell lines using subcutaneous athymic mouse models. As seen in Fig. 2C, mice receiving KMT2D-deficient cell lines had overall longer survival as these cells also had diminished capacity to propagate in vivo when compared to the parental cell line. These results, together with those described above and previous findings from the HeLa cell line [29], suggest that KMT2D inactivation compromises cell proliferation capacity in a wide range of human neoplastic cellular contexts.

### KMT2D deficiency attenuates cell migration

**Figure 3:**
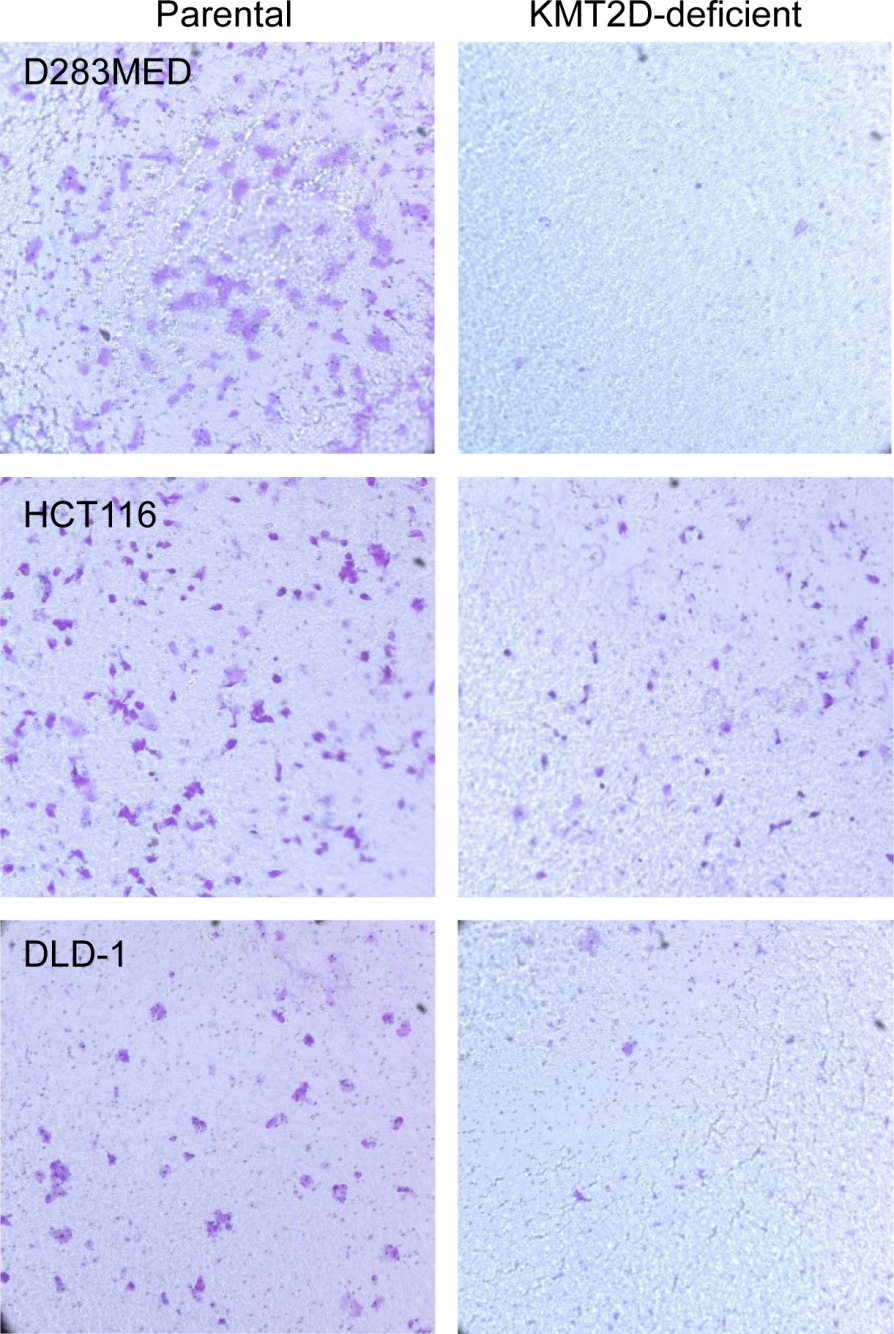
KMT2D deficiency attenuates cell migration Transwell assays were performed for D283MED, HCT116 and DLD-1 cell lines. Equal numbers of both parental and KMT2D-deficient cells were plated out in the transwell upper chamber; cells were allowed to migrate for 16 hours before those on the destination side were fixed and stained with crystal violet. Representative results from one of three independent experiments were shown. The parental D425MED cell line failed to migrate in the transwell, possibly due to its suspending nature, and was not included in this assay.

Aberrant cell migration is frequently associated with cancers. In general, enhanced migration capacity is believed to be associated with tumor metastasis. On the contrary, compromised cell migration may also make cells more susceptible to transformation. The latter is best illustrated by medulloblastoma tumorigenesis in which granule neuronal precursors fail to migrate to their destination over time and therefore acquire additional time for mitosis and are more likely to be transformed [37-39]. Our previous experiments identified a group of KMT2D-regulated genes that are functionally linked to cell migration and extracellular matrices [35]. In addition, in HeLa cells, shRNA-mediated knockdown of KMT2D altered cell cytoskeleton structure and adhesion [29]. To determine if cell migration is generally regulated by KMT2D, we performed transwell assays for pairs of isogenic cell lines. As shown in Fig. 3, when allowed 16 hours for migration, parental D283MED, HCT116 and DLD-1 cells were able to migrate through transwell chamber inserts. In contrast, the migration of KMT2D-deficient cells to the destination was attenuated. These results demonstrate that KMT2D inactivation attenuates neoplastic cell migration. Supporting this compromised cell migration are results from previous gene expression profiling in *KMT2D^−/−^* HCT116 that revealed reduced expression of genes involved in cytoskeleton organization and calcium signaling, including the cluster of S100A family genes [35].

### KMT2D regulates H3K4 monomethylation and is associated with enhancer elements

**Figure 4:**
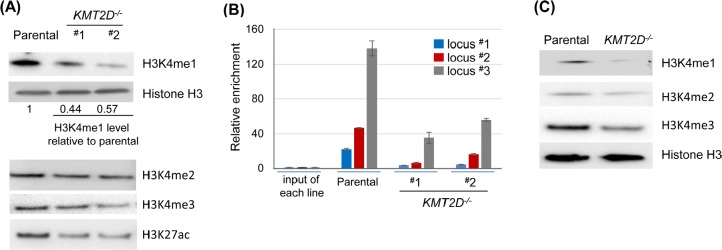
KMT2D regulates H3K4 monomethylation and is associated with enhancer elements (A) Immunoblot analysis of histone modifications in the parental versus *KMT2D^−/−^*HCT116 cell lines. Representative results from one of at least three independent experiments were shown. The relative level of H3K4me1 was indicated; the level in the *KMT2D^−/−^* cell lines was typically between 40–60% of the level in the parental cells; other modifications had slight or no reduction. (B) Anti-H3K4me1 ChIP was performed and the H3K4me1 levels were examined in a subset of KMT2D-bound enhancer elements (marked by high H3K4me1 and low H3K4me3 (ENCODE), and located >1 kilobases (kbs) upstream of the transcriptional start sites (TSS) of the closest genes). Genes associated with locus #1–3 (and the distance between their TSS and the putative enhancer elements) were *CARD10* (5.4 kbs), *WBSCR27* (8.1 kbs) and *TRIM25* (2.1 kbs). (C) Anti-H3K4me1, H3K4me2 and H3K4me3 immunoblots in the isogenic pair of DLD-1 cell lines. Representative results from one of three independent experiments were shown.

Because KMT2D functions as a histone methyltransferase, we examined the global histone modification profiles in KMT2D-null cells. In particular, given their link to the KMT2D complex, we examined the H3K4me1, H3K4me2, H3K4me3 and H3K27ac in the *KMT2D^−/−^* HCT116 cell lines compared to the parental lines. Notably, the level of H3K4me1 displayed the greatest reduction in the *KMT2D^−/−^* cell lines, while levels of other modifications only displayed minor changes (Fig. 4A). As H3K4me1 is primarily associated with global enhancer elements [40], these results prompted us to further examine the potential link between KMT2D and enhancers. We have previously mapped the global binding profile of KMT2D and revealed a large number of KMT2D bindings sites that were located outside of proximal promoter regions [35]. To further probe the link of KMT2D to enhancers, we used a stringent criteria to select 1605 KMT2D binding sites in the HCT116 cell line—identified in our previous study—and compared these loci to the HCT116 cell line histone modification profiles identified by the ENCODE project (http://genome.ucsc.edu/ENCODE/) [35, 41] (Table S1). We found that ~87% (1403/1605, p<0.0001) of KMT2D binding sites overlapped with regions positive for H3K4me1, an enhancer mark, and ~75% (1207/1605, p<0.0001) of binding sites overlapped with regions positive for H3K4me1 and H3K27ac, an active enhancer mark (Table S1). In fact, a comparison of the chromatin immunoprecipitation-sequencing (ChIP-seq) peaks of 19 transcriptional factors from ENCODE found that KMT2D had the highest fraction of binding sites that overlapped with H3K4me1 positive regions in HCT116 cell line (Table S2). These results support the link between KMT2D and H3K4 monomethylation and suggest that KMT2D frequently resides in enhancer elements. To further confirm these results, we examined the level of H3K4me1 on a set of KMT2D-bound putative enhancer loci in *KMT2D^−/−^* HCT116 cells. Notably, ChIP-qPCR revealed a reduced level of H3K4me1 in these loci in the *KMT2D^−/−^* cell lines when compared to the parental cell line (Fig. 4B). These results suggest that KMT2D resides in and regulates a set of enhancers. In agreement with this finding, another group recently used one of the *KMT2D^−/−^* HCT116 cell lines that we generated and demonstrated a similar link of KMT2D to H3K4 monomethylation and global enhancer elements [42].

KMT2C and KMT2D have similar structural domains, and prior studies suggest similar biochemical functions [34, 36]. We sought to determine if the above global reduction of H3K4me1 in the *KMT2D^−/−^* cell line was due to the loss of both KMT2D and KMT2C in the *KMT2D^−/−^* HCT116 cell line, which harbors an endogenous homozygous *KMT2C* frameshift inactivating mutation [10, 35]. Human colorectal cancer cell line DLD-1 has the wild-type *KMT2C* gene [10]. We therefore examined the H3K4me1 level in the KMT2D-null isogenic DLD-1 cell line. Interestingly, *KMT2D* knockout in DLD-1 also resulted in a reduction in the level of H3K4me1 (Fig. 4C). This result suggests that the *KMT2D* gene has a unique role in maintaining global H3K4me1 level, and that KMT2C and KMT2D may have non-redundant roles in certain cellular contexts.

### The enzymatic SET domain of KMT2D is required for effective H3K4 monomethylation in vivo

**Figure 5:**
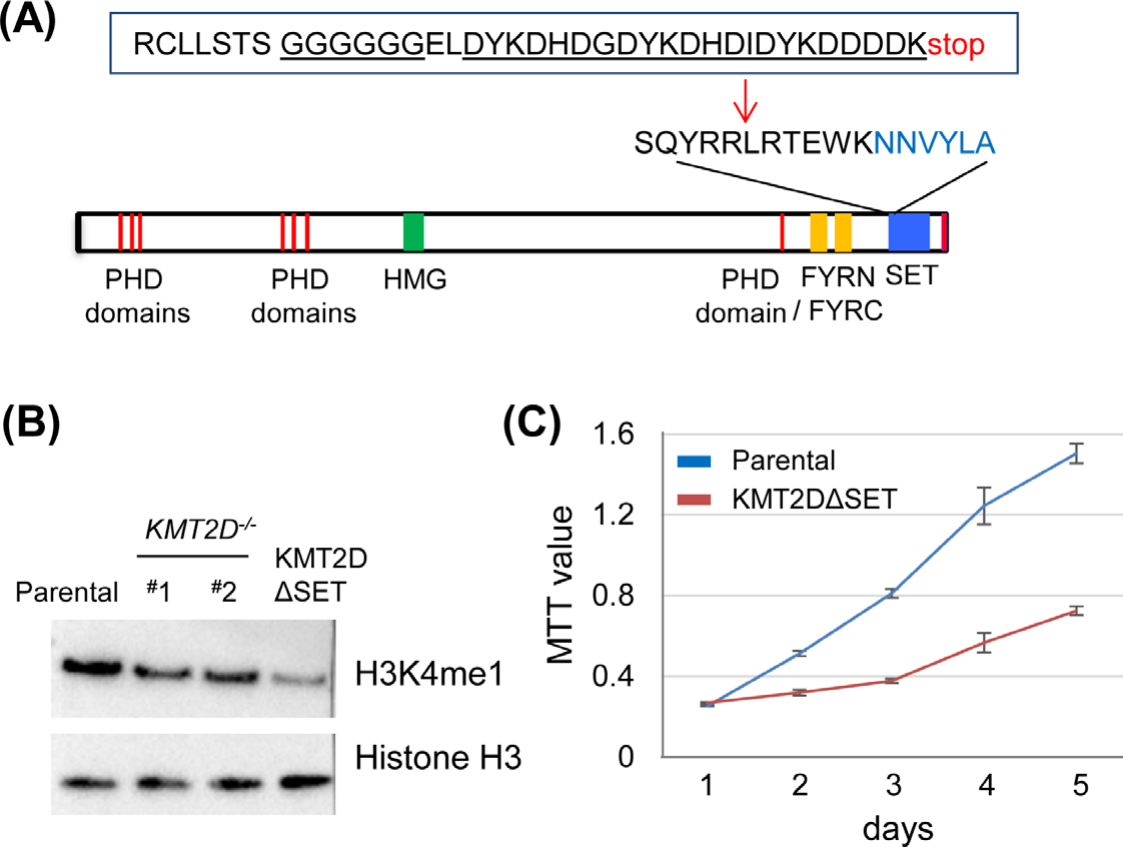
The enzymatic SET domain of KMT2D is required for effective H3K4 monomethylation in vivo (A) The design of the SET domain truncated mutant, KMT2DΔSET. The structural domains/motifs of KMT2D denoted the carboxyl-terminal enzymatic SET domain. An insertion, including a six-glycine linker and the triple Flag tags, was shown. (B) Anti-H3K4me1 Immunoblot in the isogenic set of HCT116 cell lines. Note the reduced H3K4me1 level in the KMT2DΔSET expressing cell line. (C) Proliferation of KMT2DΔSET expressing HCT116 cell line and the parental HCT116 cell line was measured by MTT assays. Representative results (from triplicate wells) from one of three experiments was shown (*p*<0.001).

The *KMT2D^−/−^* HCT116 cell lines had homozygous insertions in both *KMT2D* alleles that resulted in altered splicing transcripts with no detectable KMT2D polypeptides [35]. Despite this KMT2D-null genotype, the recent study using our *KMT2D^−/−^* cell line named this line as a KMT2DΔSET mutant, implying a simple loss of the SET domain [42]. To determine the direct role of the enzymatic SET domain of KMT2D in vivo, we generated a cell line carrying homozygous SET domain-truncated *KMT2D* alleles. Specifically, we modified our endogenous gene fusion strategy such that a six-glycine linker coding sequence, a triple-Flag coding sequence and a stop codon (gFlag: glycine-linker plus Flag) were inserted into the genome preceding the SET domain coding sequence in the *KMT2D* gene (Fig. 5A). We found that this modification overcame the previous alternative splicing issue and the expected fusion transcript and KMT2DΔSET-gFlag polypeptide (simplified as KMT2DΔSET hereafter) could be detected in this cell line (*KMT2DΔSET^gFlag/gFlag^*) (Fig. S1C). While the chromatin binding of the KMT2DΔSET mutant was detectable by ChIP, we found that the removal of the SET domain of KMT2D also led to a reduced level of H3K4me1 (Fig. S1C, Fig. 5B). Further supporting the important role of the SET domain in vivo, retarded cell proliferation was also observed in the KMT2DΔSET expressing cell line (Fig. 5C). Together, these results support the notion that the enzymatic activity of KMT2D is directly involved in regulating H3K4me1 and maintaining neoplastic cell proliferation.

## DISCUSSION

Chromatin remodeling and histone modulators are frequently altered and the resulting aberrations are associated with numerous types of cancers. The apparent loss-of-function alterations, including frameshift and nonsense mutations, suggest a tumor suppressor role for KMT2C and KMT2D. Intriguingly, knockdowns of KMT2C and KMT2D, each in a single cell type, yielded contradictive results in term of their impact on cancer cell proliferation [3, 29]. Further adding to this complexity, the near absence of homozygous *KMT2D* mutations in medulloblastoma led to the speculation of an oncogenic dependency on KMT2D activity [16]. In an attempt to clarify the role of KMT2D in neoplastic cell proliferation, we characterized a panel of isogenic human cell lines, and provided evidence to support the notion that indeed, an inactivation of KMT2D may be detrimental to the ability of certain types of neoplastic cells to propagate. These findings suggest KMT2D has distinct and complicated impacts on normal versus cancer cells. While its loss in normal cells most likely leads to, or predisposes cells to, transformation, its activity may be beneficial to the proliferation of already-transformed cells. With the ever expanding list of new cancer genes, it becomes apparent that unlike most classic cytosolic oncogenic signaling cascades, those “nucleus-originated” aberrant events, such as chromatin remodeling and histone modifications, present a different type of challenge and complication. The biological findings presented here highlight such a complexity. Similarly, the link between KMT2D to neoplastic cell migration and adhesion revealed here and in the previous study raise more questions [29]. Although we identified differential expression of numerous genes relevant to regulating cell migration, it is unclear whether simply restoring the expression of these genes can reverse the migration phenotype. The challenge is best exemplified by the S100A family of genes, as it will be difficult to overexpress a cluster of genes simultaneously. Separately, the aberrant cytoskeleton structure in the KMT2D knockdown cells raises a different set of questions [29]. It is likely this can be caused by altered expression of certain genes; but we cannot rule out the possibility of transcription-independent events such as differential post-translational protein modification. Finally, it is unclear if the KMT2D-dependent adhesion/migration is also true in normal cells, and if so, then what is its relevance to tumorigenesis.

Perhaps the most interesting finding is the revelation that KMT2D resides in and regulates enhancer elements in human cells. Our previous study identified genes regulated by KMT2D through the proximal promoter-based mechanism. The linkage of KMT2D to enhancer elements here provides a critical step toward understanding its regulatory mechanisms and its association to various types of cancers, as enhancer elements play essential yet diverse roles in cell type-dependent gene transcription [43]. This finding is in agreement with the enhancer-associated role of the Drosophila homolog gene, *Trr* [44]. Furthermore, our finding in cells harboring a SET domain truncated KMT2D mutant supports that KMT2D functions as a methyltransferase that adds monomethylation to H3K4, thereby playing a role in defining enhancer elements. It is notable that the finding in DLD-1, which, unlike HCT116, has functional *KMT2C* alleles, suggests that KMT2C cannot fully substitute for KMT2D in this cellular context. Further studies involving more cell types and in vitro enzymatic activity assays will clarify any distinct roles between KMT2C and KMT2D.

Enhancer elements have increasingly been recognized as critically involved in pathogenesis, as best exemplified by several recent studies [45, 46]. The implication of KMT2D-enhancer link to our continuing research is apparent in the following ways: first, it will guide the search of KMT2D-regulated genes through not only promoter and proximal regulatory elements but also enhancer and distal element identification; second, it suggests the role of enhancer-mediated transcriptional regulation in tumorigenesis and prompts us to examine the possible roles of other histone modifiers in regulating enhancers in both normal development and in pathogenesis; and finally, it suggests a necessity to revisit focal copy number variations in cancer cells, especially copy loss, as it is possible that the aberrant KMT2D pathway promotes tumorigenesis not just through *KMT2D* mutations, but also through the loss of its target elements.

Exploiting epigenetic aberrations for cancer treatment has been recognized as feasible, with various therapeutic agents being used in clinics or in different stages of development [47]. While the underlying mechanism remains to be illuminated, the effect of a KMT2D deficiency in neoplastic cells' proliferation supports that, in some cases, targeting KMT2D may be a viable treatment strategy, a notion recently postulated based on genetic evidence [16]. For example, it is conceivable that targeting KMT2D may serve as part of a combinatory treatment regimen, provided that a cancer-specific delivery is possible.

## METHODS

### Cell lines and somatic gene targeting

HCT116 cell lines were described previously [35]. The DLD-1 cell line (ATCC, CCL-221) was cultured in RPMI-1640 medium supplemented with 10% FBS. Medulloblastoma cell lines D425MED and D283MED were cultured in zinc option medium (Life Technologies, cat# 10373017; supplemented with 10% FBS). The rAAV-mediated gene knockout was described previously [35]. For ZFN-mediated gene knockout, *KMT2D*-specific ZFN expression plasmids (Sigma, cat# CKOZFND14397) were used to transfect cells in 6-well plates. Two days after transfection, cells were serially diluted, plated out in 96-well plates and cultured for 10–14 days before screening for *KMT2D*-mutant clones using Cel-I assays. Positive clones were sequenced and those with frameshift mutations were defined as null mutations and used for further studies. For homozygous *KMT2D* knockout in DLD-1, the 1^st^ allele was knockout by ZFN and the 2^nd^ allele was knockout by the previously described rAAV-mediated strategy [35]. Homozygous knockout was confirmed by anti-KMT2D immunoblot using the antibody previously described [35].

### Nuclear extraction, immunoblot, chromatin immunoprecipitation (ChIP) and quantitative PCR

Procedures for these experiments were previously described [35]. Antibodies used for ChIP and for immunoblot included: anti-H3K4me1 (Millipore, cat#07-436), anti-H3K4me2 (Abcam, Cat#: ab7766), anti-H3K4me3 (Millipore, cat#: 07-473), and anti-H3K27ac (Millipore cat# 05-1334). Relative H3K4me1 levels were quantified using ChemiDoc MP Imaging System (Bio-Rad). Anti-Flag antibody for anti-Flag ChIP was previously described [35]. The same protocol was used for anti-H3K4me1 ChIP, except that the cells were cross-linked with 1% formaldehyde and sonicated with a Branson sonifier only. PCR primers used for ChIP-qPCR included: locus #1 (*CARD10*): forward primer 5′-CAGGCTGGGAAAAGACAGAG-3′ and reverse primer 5′-CTCCCTCCAACCACCTTGT-3′; locus #2 (*WBSCR27*): forward primer 5′-GCTGACACACCCAGCTGATA-3′ and reverse primer 5′-ACCCTCCAGTGGGTTAAAGC-3'; locus #3 (*TRIM25*): forward primer 5′-GTGCAATAGGCATGCAACAG-3′ and reverse primer 5-GGACCTGACTTCTCCCCATA-3′. Primers for other ChIP-qPCR were previously described [35].

### Comparison of KMT2D binding sites to regions identified by ENCODE

KMT2D binding sites (Table S1) were from previous data analyzed by model-based analysis of ChIP-Seq (MACS) [35, 48]. ENCODE data for H3K4me1, H3K27ac and other transcriptional factors in the HCT116 cell line were downloaded from http://genome.ucsc.edu/ENCODE [41]. Comparative analysis of the ENCODE data and KMT2D-Flag binding sites was performed using the ChIPpeakAnno package from the R software [49]. For statistical analysis of the overlap between KMT2D loci and H3K4me1 or H3K4me1/H3K27ac loci, 10,000 sets of equal numbers (1605) of loci with comparable widths across the genome were randomly generated and their overlapping with H3K4me1 or H3K4me1/H3K27ac loci was tested.

### MTT assays and colony formation assays

For the MTT cell proliferation assay, cells were plated out in 96-well plates at a density of 1×10^4^/well in 200 μl medium. 20 μl MTT (Thiazolyl Blue Tetrazolium Bromide, 5 mg/ml, Sigma-Aldrich, cat# M5655) was added to each well and incubated for 3.5 hours. MTT solvent (4 mM HCl, 0.1% Nondet P-40 (NP40) in isopropanol) was used to dissolve the pellets. Plates were read at 595nm. For D425MED, D283MED and HCT116 cell lines, the result from one representative experiment was shown. OD values from triplicate wells were presented as mean±sd. For DLD-1 cell line, the result from three independent experiments was shown. P value was calculated by unpaired t test. For the plate colony formation assay of HCT116 and DLD-1 cells, 500 cells in single cell suspension were plated out into each well of 6-well plate. Cells were allowed to grow for 12–14 days, then fixed with 1% formaldehyde and stained with 0.05% crystal violet solution, For medulloblastoma cells, a soft agar colony formation assay was performed. Briefly, 0.5% agar was used as the base agar and 1×10^3^ cells in 0.25% top agar was plated in each well of a 6-well plate. Colonies were counted after 14 days. For D425MED cell lines, the result from one representative experiment (triplicate wells) was shown. P value was calculated by unpaired t test.

### Cell migration assays

Transwell chamber assay was used to examine migration of the cells. 24-well plates with 6.5 mm inserts (8.0 μm pore size) (Corning, cat# 3422) were used. The inserts were pre-coated with 1% gelatin. 1×10^5^ cells in serum-free medium were added to the upper chamber. The lower chamber was filled with complete medium (10% FBS). After 16 hours of incubation, the medium in the top chamber was removed. Cells on the upper surface of the inserts were wiped off with swabs. Cells on the lower surface of the inserts were stained with 0.5% crystal violet.

### Tumorigenicity analysis in xenograft models

All experiments were approved by the Animal Care Committee of the Duke University Medical Center. Facility-bred athymic BALB/c nu/nu nude male mice, 4–6 weeks of age, were maintained at the Cancer Center Isolation Facility (CCIF) of the Duke Cancer Institute. Cancer cell lines were grown to 60–80% confluence, harvested, washed with PBS, and injected subcutaneously (1×10^7^ cells per mouse) into the right flanks. Tumors were measured for their widths and lengths using a micro-caliper. Tumor volumes were calculated (width^2^ × length/2). Tumors were first measured at day 4 post-implantation, and twice per week afterward. Survival endpoint was defined as the day when the tumor volume reached at least 1000 mm^3^ and at least five times the volume of the initial measurement. A Kaplan-Meier survival curve was plotted using SPSS.

## Supplemental Figures and Tables




