# Supplementary material for: KMT2D maintains neoplastic cell proliferation and global histone H3 lysine 4 monomethylation
Source: Oncotarget. 2013 Nov 3;4(11):2144–53. doi: 10.18632/oncotarget.1555 (PMC3875776; doi:10.18632/oncotarget.1555)
Supplement: Supplementary file 1 [file oncotarget-04-2144-s001.pdf]

KMT2D maintains neoplastic cell proliferation and global histone H3 lysine 4 monomethylation - Guo et al

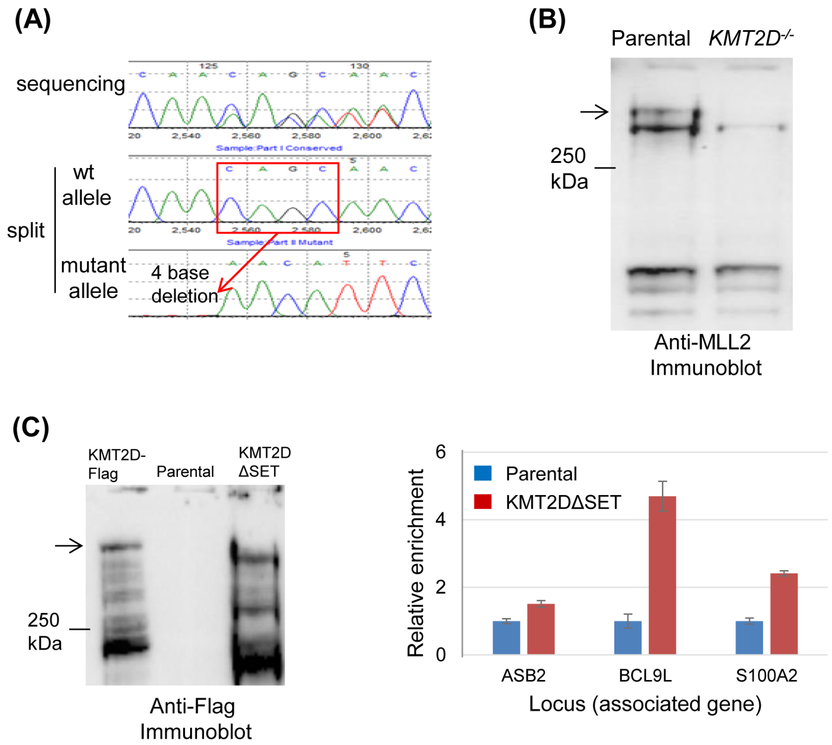

Figure S1: Somatic gene editing and gene knockout in human neoplastic cell lines. (A) An example of somatic *KMT2D* gene knockout by ZFNs. Sequencing of the *KMT2D* gene identified a D425MED cell line with heterozygous frameshift mutation, a deletion of four bases. (B) Anti-KMT2D immunoblot identified an isogenic DLD-1 cell line that was KMT2D-null. The arrow points to the expected KMT2D polypeptide. (C) Left: Anti-Flag immunoblot confirmed the expression of KMT2DΔSET polypeptide; Right: Anti-Flag ChIP coupled with quantification PCR confirmed the detectable binding of KMT2DΔSET to the expected KMT2D-targeted loci.
